# Supplementary material for: Association between oral microbiome and seven types of cancers in East Asian population: a two-sample Mendelian randomization analysis
Source: Front Mol Biosci. 2023 Nov 21;10:1327893. doi: 10.3389/fmolb.2023.1327893 (PMC10702768; doi:10.3389/fmolb.2023.1327893)
Supplement: Supplementary file 1 [file DataSheet1.ZIP › Supplementary Materials/MR plots for tongue/tongue═╝/Ovarian cancer/pheno.473_to_ovarian cancer_leave_one_out.pdf]

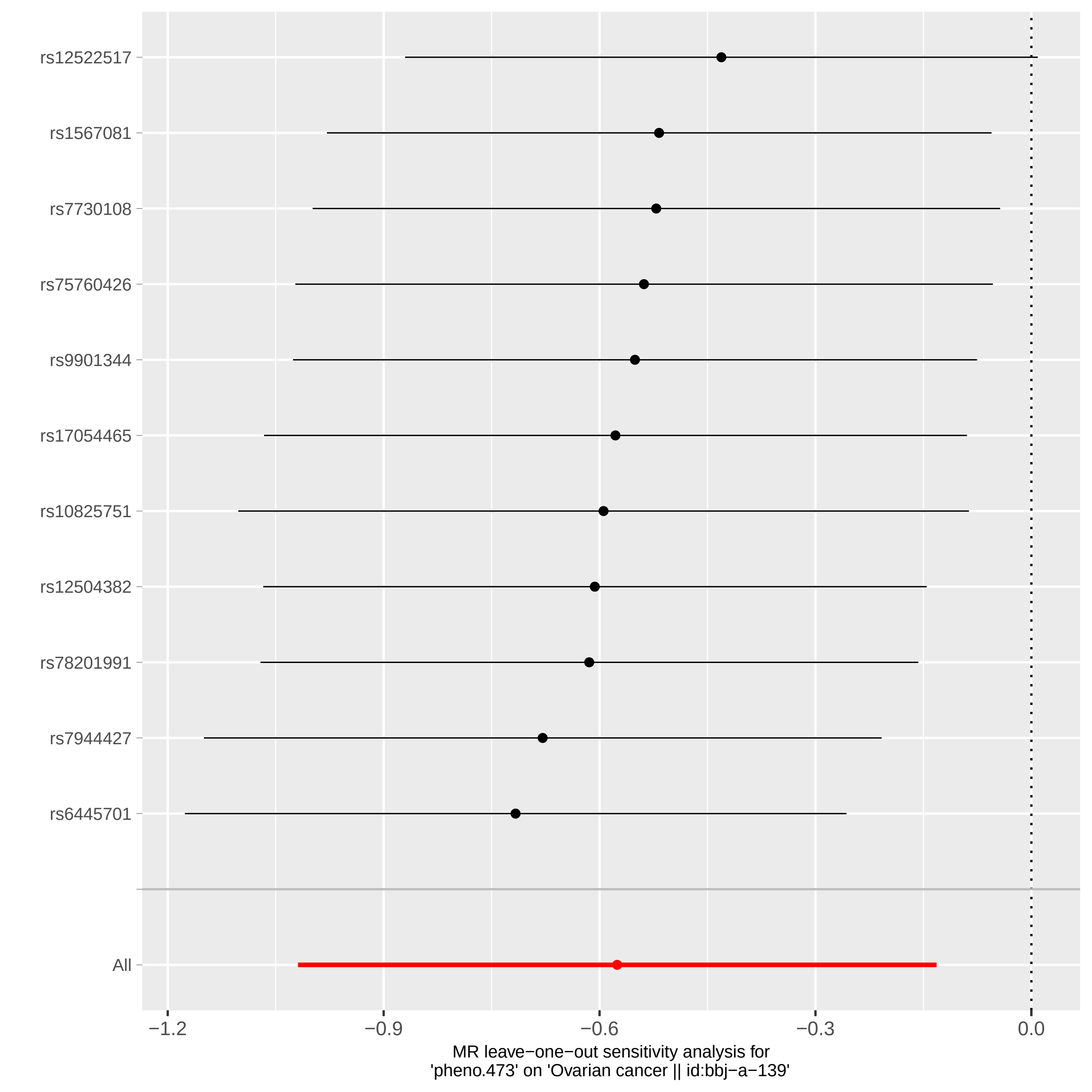

rs12522517
rs1567081
rs7730108
rs75760426
rs9901344
rs17054465
rs10825751
rs12504382
rs78201991
rs7944427
rs6445701
All
−1.2
−0.9
−0.6
−0.3
0.0
MR leave−one−out sensitivity analysis for
'pheno.473' on 'Ovarian cancer || id:bbj−a−139'
